# Mr. Charles Ralph Keyser

**Published:** 1910-07-09

**Authors:** 


Mr. Charles Ralph Keyser, aged thirty-six, has died at sea on board s.s. *Athenic*. He studied at St. George's Hospital, took his diploma in 1896, and became a F.R.C.S. in 1899. His appointments included those of surgeon to the Cancer Hospital and consulting surgeon to the Eliot Cottage Hospital, Hayward's Heath. At St. George's Hospital Mr. Keyser was at different times demonstrator of anatomy and operative surgery, house surgeon, house physician, and surgical registrar. He also held the post of clinical assistant to the Victoria Hospital for Children, Chelsea.

